# Characteristics of Physician Empathetic Statements During Pediatric Intensive Care Conferences With Family Members: A Qualitative Study

**DOI:** 10.1001/jamanetworkopen.2018.0351

**Published:** 2018-07-06

**Authors:** Tessie W. October, Zoelle B. Dizon, Robert M. Arnold, Abby R. Rosenberg

**Affiliations:** 1 Department of Pediatrics, George Washington University School of Medicine, Washington, DC; 2 Division of Critical Care Medicine, Children’s National Health Systems, Washington, DC; 3 Section of Palliative Care and Medical Ethics, Division of General Internal Medicine, University of Pittsburgh Medical Center, Pittsburgh, Pennsylvania; 4 Division of Hematology-Oncology, Department of Pediatrics, University of Washington School of Medicine, Seattle; 5 Division of Bioethics-Palliative Care, Department of Pediatrics, University of Washington School of Medicine, Seattle; 6 Seattle Children’s Research Institute, Center for Clinical and Translational Science, Treuman Katz Center for Pediatric Bioethics, Seattle, Washington

## Abstract

**Importance:**

Pediatric intensive care unit care conferences often involve high-stakes decisions regarding critically ill children, resulting in strong family emotions. Families often report the need for physician empathy.

**Objective:**

To evaluate the characteristics of physician empathetic statements during pediatric intensive care unit care conferences.

**Design, Setting, and Participants:**

In this single-center, cross-sectional, qualitative phenomenology study, 68 transcripts of audio-recorded care conferences were analyzed from an urban, quaternary medical center from January 3, 2013, to January 5, 2017. Thirty physicians and 179 family members of 68 children participated in care conferences. Data analysis was conducted from June 5, 2017, to October 12, 2017.

**Main Outcomes and Measures:**

A qualitative thematic analysis was conducted to code physician empathetic statements and family’s responses to these statements. Empathetic statements were classified using the previously published NURSE pneumonic (naming, understanding, respecting, supporting, exploring) and coded as unburied (statement followed by a pause allowing the family time to respond) or buried (empathetic statement encased in medical talk or terminated with a closed-ended statement). Family responses were categorized into 3 themes: alliance (emotion continued), cognitive (medical talk), or none. Missed opportunities for physicians to respond with empathy were identified.

**Results:**

Thirty physicians participated, of whom 13 (43%) were male, 24 (80%) were white, 24 (80%) had more than 5 years of practice, 10 (33%) specialized in critical care, and 7 (23%) specialized in hematology/oncology. Within 68 care conferences, physicians recognized families’ emotional cues 74% of the time, making 364 empathetic statements. Of these statements, 224 (61.5%) were unburied and 140 (38.5%) were buried. Buried statements were most commonly followed by medical talk (133 [95.0%]). Unburied empathetic statements were associated with alliance responses from the family 71.4% of the time compared with 12.1% of the time when the statement was buried (odds ratio, 18; 95% CI, 10.1-32.4; *P* < .001). Physicians missed an opportunity to address emotion 26% of the time, with at least 1 missed opportunity occurring in 53 conferences (78%). Physicians attended to all family emotions in only 5 conferences (7%).

**Conclusions and Relevance:**

In this analysis, physicians responded with empathy frequently, but responses were buried within other pieces of medical data or missed entirely in nearly one-third of conferences. When physicians responded using unburied empathetic statements and allowed time for family members to respond, they were more likely to learn important information about the family’s fears, values, and motivations.

## Key Points

**Question:**

When physicians attend to family emotions, how does it influence ensuing communication?

**Findings:**

In this qualitative study of 68 pediatric intensive care conferences with 179 family members, physicians recognized family emotions 74% of the time and missed opportunities to respond to emotion 26% of the time. When physicians’ empathetic statements were followed by a pause, rather than additional medical talk, family-participants were 18-fold more likely to share concerns, hopes, or values.

**Meaning:**

When physicians attend to emotions and then stop, family-participants have time to express emotions and provide valuable information.

## Teaser

This qualitative study characterizes the frequency and nature of physician empathetic statements during pediatric intensive care unit (PICU) care conferences with families, the families’ responses to the statements, and missed opportunties for communication.

## Introduction

Care conferences between physicians and families of critically ill children in the pediatric intensive care unit (PICU) are often convened to review a patient’s condition, discuss prognosis, and/or make an important medical decision.^1,2,3^ Most often, care conferences are offered when a patient’s clinical condition has worsened or when there is a need for medical decisions about the plan of care.^1^ Because these meetings typically involve high-stakes decisions, such as whether to limit life-sustaining therapies, families are under extreme stress and emotions are strong.^4,5,6^ Studies have shown families experience powerful positive and negative feelings during their time in the intensive care unit (ICU).^6,7,8^ These may be further heightened when discussing serious news or facing life-changing decisions.

Families have consistently reported a desire for their physicians to show empathy.^9,10^ Empathy is a vital component of high-quality health care,^11,12^ and physician statements of empathy are an important source of support for patients and their families.^13^ Literature from adult ICUs suggests physicians infrequently show empathy^14^ and often miss opportunities to connect with families.^15^ We do not know how often pediatricians show empathy or how expressions of sentiments affect communication during care conferences. Our objectives were to evaluate the characteristics of physician empathetic statements during family care conferences in the PICU. We also explored if and when these opportunities were missed.

## Methods

### Study Design and Setting

We conducted a single-center, cross-sectional, qualitative phenomenology study analyzing 68 transcripts of family-physician care conferences in the PICU. Transcripts were collected from January 3, 2013, to January 5, 2017, from an urban, quaternary medical center with a 44-bed mixed medical and surgical PICU, excluding patients with primary cardiac conditions, who were cared for in a separate cardiac ICU. This study was approved by the Children’s National Health System institutional review board, and written informed consent was obtained from all participants in the care conference.

### Data Collection

We defined a care conference as a scheduled meeting between the family of a critically ill child (newborn to 26 years) and the PICU attending physician or primary consultant. In our PICU, care conferences are convened at the discretion of the PICU attending physician or by family request.

Eligible care conferences included those in which the PICU attending physician or primary consultant anticipated discussing a medical decision with an English-speaking family of a critically ill child. Medical decisions were defined as decisions to initiate, escalate, or withdraw medical interventions, such as endotracheal tubes, extracorporeal membrane oxygenation, surgical procedures, and discussions of resuscitation status. We only included weekday care conferences, which was when primary care team members were most available. We excluded conferences with the intention to discuss discharge planning or provide a medical update because our primary goal was to assess physician use of empathy during high-stakes, decision-making care conferences.

To identify care conferences, study personnel contacted PICU attending physicians each weekday morning to determine if they anticipated conducting a care conference. After screening for eligibility, study personnel consulted with the care team (ie, clinical nurse, social worker, and attending physician) to determine if there were reasons this particular family should not be approached. Families were eligible to participate once in the study; attending physicians could be enrolled up to 4 times. Each care conference was audio recorded and transcribed verbatim, with personal identifiers removed prior to analysis. We collected demographic data from families via surveys and clinical data from electronic medical records. We also collected demographic data from the physicians via surveys, including years of practice, medical specialty, sex, and race.

### Data Analysis

Our primary outcome measure was to conduct a qualitative thematic analysis following Standards for Reporting Qualitative Research (SRQR) reporting guidelines to code physician empathetic statements in care conferences and the family’s response to these statements. We also analyzed empathetic statements made by other members of the health care team. We applied directed content analysis^16^ to code the empathetic statements and responses. We used investigator triangulation,^17^ with 3 investigators (T.W.O., Z.B.D., and A.R.R.) reviewing all transcripts and 2 reviewing all codes. For context, we represented pediatric clinicians with experience in critical care (T.W.O.), oncology (A.R.R.), palliative care (T.W.O. and A.R.R.), and investigators with formal training in health services and qualitative research (T.W.O., Z.B.D., and A.R.R.). Additionally, Dr October may have had direct clinical contact with participants as one of their attending physicians, although she was neither in that role nor present for any of the recorded care conferences.

Any discrepancies in coding were resolved through discussion until a consensus was reached. A random sample of 20% of the comments was then selected by a third researcher to determine interrater reliability between the 2 coders; Cohen κ was 0.85 (*P* < .001). Quantitative data were analyzed using SPSS Statistics software version 24 (IBM).

An empathetic statement was defined as any statement made by a physician that attempted to convey the physician understood or shared the family’s emotional state. A family’s emotional state was defined as statements expressing negative emotion, such as sadness, anger, frustration, or helplessness, eg, “Seeing her lying there, not being able to comfort her, I feel so helpless.” We used the NURSE pneumonic (naming, understanding, respecting, supporting, exploring)^13,18^ to identify and classify the empathetic statements into 5 categories: naming the family’s emotion, understanding the family’s emotional response, respecting the family‘s expert role in the child’s care, supporting the family’s needs and/or decisions, and exploring the family’s feelings, concerns, or hopes. For example, if a family member hears upsetting news and says, “I am so upset I wasn’t there,” an empathetic response from the physician of, “You have been such a good parent to your child. Always at his bedside,” would classify as a respect NURSE statement.

Empathetic statements were coded as unburied or buried. Unburied statements were defined as empathetic statements made by the physician followed by a pause to allow the family time to respond. Contrarily, buried statements were defined as empathetic statements made by the physician and obscured in 1 of the following ways: (1) medical talk, which was (a) encased in more than 2 sentences of medical text; (b) linked with “but” and followed by more medical talk; or (c) followed by a second physician speaking, not allowing time for a family response; or (2) closure by the physician, who asked a closed-ended question.

We collected the family’s response to the physician’s buried or unburied empathetic statement and categorized them based on emergent themes. Family responses fell into 3 themes: an alliance response, a cognitive response, or no response (Table 1). Alliance responses were defined as statements that deepened the emotional discussion, expressed gratitude, demonstrated agreement, or expressed mourning. Cognitive responses were defined as statements in which the family asked a medical question or continued to talk about medical information, ignoring the emotional statement. No response occurred when the conversation ended without a family response either because the physician asked a closed-ended question or a member of the health care team interrupted, preventing the family from responding to the emotional statement.

**Table 1.** Thematic Categories of Family Responses to Physician Empathetic Statements

Category of Family Response	Definition of Category	Sample Quote^a^
Alliance response		
Deepen emotional discussion	Family shares their hopes and fears	Physician: *This is not making sense, I understand. It is no one’s intent to have such a thing happen…and it’s a nightmare.* Family: It is, and it’s never going to end because my son won’t be leaving out here with me. I don’t know what to do.
Express gratitude	Family expresses thanks for the care or concern from the physician	Physician: *He’s so lucky to have you guys…he has an amazing family—you all are amazing.* Are there any questions we haven’t answered? Family: No questions. I really, really appreciate it. He wouldn’t have survived without you, so I really appreciate the doctors and the hospital.
Agreement with physician	Family statement aligns with the physician’s statement	Family: I agree. I think that talking to the tracheostomy nurse would be a good idea.
Express mourning	Family becomes tearful or asks for a moment to collect themselves	Family: [family crying]
Cognitive response	Family asks a medical question or makes a statement continuing the medical talk, ignoring the empathetic statement	Physician: *Believe me, we want to do that. We want to do what’s best for him as fast as possible.* We thought about removing the kidneys first, but it’s a big surgery. He won’t make any urine anymore. His kidney function is declining, but still the kidneys are doing at least a little bit, and that helps even a little bit. So that’s why we want to try this peritoneal dialysis first. Family: That would be a drastic change, removing the kidney.
No response	Conversation ends without a response from the family because the physician asked closed-ended question or a member of the health care team interrupted prior to a family response	Physician: *I think you guys did a great job. You expressed your thoughts very clearly and we had a good 2-way conversation.* We’ll come in separately for the week and touch base with you. Alright? Family: Mmhmm.

^a^ The empathetic physician statement is italicized in the sample quotes.

Differences between family responses to physician buried vs unburied empathetic statements were calculated using Pearson χ^2^ analysis with a 2-sided α of .05, and odds ratios with 95% confidence intervals are presented. Differences between medical specialty, physician sex, or number of years of practice and buried vs unburied statements were calculated using Fisher exact test.

We identified missed opportunities for physicians to show empathy. These missed opportunities, as defined by Curtis et al,^15^ were classified as passages in which the physician failed to respond to a family’s emotional statements. For example, a family member may say, “This shouldn’t happen to a baby.” A physician’s response of “Let’s talk about the next steps” would classify as a missed opportunity rather than responding with an empathetic statement. If the same missed opportunity was present multiple times throughout the same passage of the transcript but was still representative of the same theme, it was collectively counted as 1 missed opportunity.

## Results

### Demographic Characteristics

We identified 88 eligible care conferences, of which 10 families declined participation because they did not consent to audiotaping (n = 3) or felt emotionally overwhelmed (n = 7). An additional 10 families were not approached based on the care team’s recommendation (eg, if there was concern for psychologic instability or if there was legal involvement), resulting in 68 audio-recorded family-physician care conferences (77% enrollment). A total of 179 family members participated in the 68 care conferences; 67 (99%) included the mother, 50 (74%) included the father, and 62 (91%) included an additional family member.

A total of 30 physicians participated. Of these, 13 (43%) were male, 24 (80%) were white, 24 (80%) had more than 5 years of practice, 10 (33%) specialized in critical care, and 7 (23%) specialized in hematology/oncology (Table 2). In the 68 care conferences, the most common decision discussed was tracheostomy placement (30 [44%]), followed by the family’s goals (13 [19%]), surgical procedure (10 [15%]), and medical treatment (9 [13%]). Of the participating patients, 43 (64%) were black, and 15 (22%) were white. They were most commonly discharged home (29 [43%]) or to a subacute facility (19 [28%]). Fifteen (22%) died during hospitalization. The care conferences’ participants included on average 2.6 family members and 6 health care team members. Aside from physicians, other health care team members included most often a social worker (62 [91%] of care conferences), a case manager (29 [43%]), or a bedside nurse (24 [35%]).

**Table 2.** Demographic Characteristics

Characteristic	No. (%)		
	Patients (n = 68)	Family Members (n = 179)	Physicians (n = 30)
Male	41 (60)	77 (43)	13 (43)
Age, median (IQR), mo	42.5 (14.8-120.0)	ND	ND
Race			
Black	43 (64)	108 (60)	2 (7)
White	15 (22)	43 (24)	24 (80)
Asian	3 (4)	6 (4)	2 (7)
Other	7 (10)	22 (12)	2 (7)
Diagnosis			
Hematologic/oncologic	15 (22)	NA	NA
Respiratory	14 (21)	NA	NA
Neurologic	13 (19)	NA	NA
Congenital/genetic	11 (16)	NA	NA
Shock/trauma	8 (12)	NA	NA
Other	7 (10)	NA	NA
Treatment decision discussed			
Tracheostomy	30 (44)	NA	NA
Overall goals of care	13 (19)	NA	NA
Surgical procedure	10 (15)	NA	NA
Medical treatment	9 (13)	NA	NA
Withdrawal of technical support	6 (9)	NA	NA
Disposition			
Home	29 (43)	NA	NA
Rehabilitation/subacute facility	19 (28)	NA	NA
Deceased	15 (22)	NA	NA
Other hospital	4 (6)	NA	NA
Hospice	1 (1)	NA	NA
Relationship to patient			
Mother	NA	67 (37)	NA
Father	NA	50 (28)	NA
Other family member	NA	62 (35)	NA
Medical specialty			
Critical care	NA	NA	10 (33)
Hematology/oncology	NA	NA	7 (23)
Neurology	NA	NA	2 (7)
Pulmonology	NA	NA	2 (7)
Genetics	NA	NA	2 (7)
Surgery	NA	NA	2 (7)
Other^a^	NA	NA	5 (17)
Years of practice, y			
<5	NA	NA	6 (20)
>5	NA	NA	24 (80)

Abbreviations: IQR, interquartile range; NA, not applicable; ND, data not collected.

^a^ Other represents 1 physician in each of the following specialties: cardiology, complex care, nephrology, physical medicine and rehabilitation, and primary care.

### Empathetic Statements

In the 68 conferences, physicians recognized families’ emotional cues 74% of the time, making 364 empathetic statements identified using the NURSE pneumonic (range, 1-15 statements per conference; mean, 2.8 statements per conference) (Table 3). Of these 364 empathetic statements, 224 (61.5%) were unburied and 140 (38.5%) were buried. Buried statements were most commonly followed by medical talk (133 [95.0%]), of which 72 (54.1%) featured the same physician continuing with medical data, 34 (25.6%) featured the same physician connecting the empathetic statement with medical talk using “but,” and 27 (20.3%) featured a second physician interrupting with more medical data. The remaining 7 buried statements (5.3%) were followed by a closed-ended question (Table 4). We did not find a relationship between whether the empathetic statement was buried or unburied and physician medical specialty, sex, or years of practice.

**Table 3.** Empathetic Statements Made by Physicians Using the NURSE Pneumonic to Respond to Family Emotions

Type of NURSE Statement	Definition	Statements, No. (%) (n = 364)	Sample Quotes
Naming	Name the family’s emotion	48 (13)	It sounds like you were anxious because you didn’t feel there was as much attention given.
Understanding	Empathize with and acknowledge the family’s emotional response	110 (30)	I completely understand. This has to be the hardest thing you’ve ever gone through.
Respecting	Praise the family for their expert role in their child’s care	90 (25)	You guys have been remarkably ahead of us in everything.
Supporting	Demonstrate support of the family’s needs and/or decisions	83 (23)	There’s no right answer here, they are just both very hard. We are here to support you. If there’s any way we can help you, we’ll be there.
Exploring	Ask the family to elaborate their feelings, concerns, or hopes	33 (9)	Dad, I’ve been talking more to Mom because she’s sitting across from me, and I apologize for that—what are your thoughts?

Abbreviation: NURSE, naming, understanding, respecting, supporting, exploring.

**Table 4.** Categories of Buried Empathetic Statements by Physicians

Category of Buried Empathy	Definition	Statements, No. (%) (n = 140)	Sample Quotes^a^
Medical talk		133 (95)	
Additional data	Empathetic statement buried within >2 sentences of medical text or information giving, such as orientation, instructions	72 (54)	Physician: *We’re here to support you* and give you our team-by-team perspective on how to take care of [patient]. So from the ICU standpoint, we are weaning his ventilator again today, and probably in the next 1 or 2 days, we’ll have him at the point where the ventilator is doing very little work and he’s going to do all the breathing on his own, still with the tube in place. At that point, we’ll trial him and do what is called an extubation trial.
But	Empathetic statement connected with a “but” to medical talk	34 (26)	Physician: No one has a crystal ball, and *all of us hope and pray for miracles to happen to people, and we support you in your hopes and your prayers*, but we also have never seen someone who has been this ill showing no signs of getting better after all this time get better.
Second physician	Consultant or other health care team member speaks next, not leaving time for silence or parent response	27 (20)	Physician 1: *I understand your frustration.* Physician 2: The beauty of the biomedical markers is that those aren’t dependent on exercise; they don’t depend on whether he’s in a good mood or if he’s sedated.
Closed	Empathetic statement followed by a closed-ended question	7 (5)	Physician: *We appreciate you sharing your thoughts and feelings about this. It’s tough. We understand that, and we appreciate that.* Well, I don’t have anything else, if no one else does—we’re here if you need us. If there’s anything else that comes up, I’m here. Okay?

Abbreviation: ICU, intensive care unit.

^a^ The empathetic physician statement is italicized in the sample quotes.

Another member of the health care team, such as the social worker or bedside nurse, was present in all 68 care conferences and spoke on average 5% of the time. In 47 care conferences (69%), they did not verbally contribute an emotional statement. In the remaining 21 care conferences (31%), nonphysician team members made 53 empathetic statements, of which 46 (87%) were unburied and 7 (13%) were buried. Using the NURSE pneumonic, 48 statements (25%) were naming, 90 (28%) were respecting, and 83 (30%) were supporting statements.

### Family Responses Based on Type of Empathetic Statement (Buried or Unburied)

When comparing family responses with unburied and buried empathetic statements, we found when physicians buried the empathetic statement, it frequently stopped the progression of the conversation and led to an alliance response only 12.1% (17 of 140) of the time (Table 5). Contrarily, when physicians made an empathetic statement and paused to allow the family time to respond, the family was able to continue to emote and led to an alliance response of 71.4% (160 of 224) of the time. The odds of a physician receiving an alliance response from families was 18-fold higher (95% CI, 10.1-32.4; *P* < .001) when the physician used an unburied statement compared with a buried statement. Buried statements also did not result in any family expressions of mourning, such as crying or asking for time to collect themselves, whereas unburied empathetic statements resulted in expressions of mourning in 14 responses (8.8%).

**Table 5.** Family Responses to Physician Unburied and Buried Empathetic Statements

Family Response Theme	No. (%)		*P* Value^a^	Odds Ratio (95% CI)
	Unburied Empathy (n = 224)	Buried Empathy (n = 140)		
Alliance response	160 (71)	17 (12)		
Deepen	95 (59)	11 (65)	<.001	18 (10.1-32.4)
Gratitude	26 (16)	1 (6)		
Agreement	25 (16)	5 (29)		
Mourning	14 (9)	0 (0)		
Cognitive response	57 (26)	69 (49)	NA	NA
No response	7 (3)	54 (39)	NA	NA

Abbreviation: NA, not applicable.

^a^ Differences between family responses to physician buried vs unburied empathetic statements were calculated using Pearson χ^2^ analysis with 2-sided α of .05.

### Missed Opportunities for Empathy

Missed opportunities were occasions when the family expressed an emotion and the physician did not respond with empathy, which occurred 125 times (26% of empathetic opportunities). At least 1 missed opportunity occurred in 53 conference (78%). In only 5 conferences (7%) were all of the family’s emotional opportunities attended to by the physician. We identified 5 categories of missed opportunities based on when physicians

(1) Responded with a medical statement (44 [35%]),

Family: I’m nervous about going home with that [chest tube]. It scares me.

Physician: At one point, we were even debating putting a chest tube in that Saturday. We all thought it was the right thing to do for [patient]. If you pull that chest tube out, he would feel short of breath. It would trap secretions into his lungs; it would set him up for infection. I think it would be painful and I think there’s a chance that it could compromise the blood flow to his body and be what we call a tension pneumothorax.

(2) Negated the family’s emotional statement by attempting to discount it as false (29 [23%]),

Family: I told them when he cries so loud and his whole body turns red and he gets very still—it really worried me. It wasn’t just the cry, it was his body language. It wasn’t often, it would just be at certain times, he would do exactly what I just said, and it worried me.

Physician: The most common reason for a baby to do that is actually reflux, especially if it happens episodically like that.

(3) Pivoted by simply moving on to another topic (29 [23%]),

Family: That’s the only option. It’s either [the tracheostomy] or he passes away and I don’t think that’s fair to him.

Physician: Do you have family down in [location]?

(4) Ignored the comment completely (20 [16%]),

Family: I feel like he’s in pain. I know you guys don’t, but I do. I know you guys might think I’m wrong, but he’s so sensitive… He was breathing on his own a little yesterday, until he started the breathing treatment, which was really rough on him. So I don’t know if he shut down because of pain.

Physician: Yeah, the breathing issues are something we’re going to have to address eventually.

(5) Deferred responsibility to another member of the health care team (3 [2%]),

Family: It’s very concerning to us. We feel someone needs to be ultimately responsible for her case…and not have 10 different hands in the pot.

Physician: I have not seen her before this. I’ve only heard and talked about her echocardiograms, but she was seen by the heart failure team, not the general cardiology consult team.

## Discussion

Expressions of emotion from families during decision-making discussions regarding a critically ill child are common, and finding the right response to a family’s emotion can be challenging. When families express emotions, physicians may recognize it and respond empathetically or they might miss or ignore the opportunity, pressing on in the delivery of medical data. We wanted to assess how these types of responses impact the flow of conversation and whether they help or hinder family-physician communication.

Like other studies,^19,20^ we found physicians responded with empathy frequently. In this study we demonstrated that how the physician responded empathetically made a difference. Importantly, in nearly half of cases where the empathy was transparent and unburied, the conversation progressed or deepened, and physicians learned new information about a family’s motivations, fears, worries, and/or hopes.

Empathy not only builds the physician-family relationship,^21,22,23^ it also helps physicians make treatment recommendations by promoting better data collection and information gathering. Value-laden decisions require input from patients and their families.^24^ In 2016, the American College of Critical Care Medicine identified preference-sensitive decisions, such as tracheostomy placement, goals of care, or withdrawal of technical support (72% of the decisions in our cohort), that should trigger physicians to explore a family’s values.^25^ Responding with unburied empathy may be an effective strategy to ensure families feel heard and present an opportunity for the physician to learn values most important to the family.

When the empathetic statement is buried, our data suggest families may not hear the physician’s attempt to connect with them empathetically. How physicians bury empathy is predictable. It was most commonly buried within complex medical talk or attached to medical statements with a “but.” *But* is a conjunction aimed at connecting ideas, and it often serves to contradict the next clause. We suspect physicians use medical talk or “but” to quickly attempt to address the emotion, then return to what is most comfortable. For families, using “but” may make that clear and lead them to move away from the emotion and stay in medical talk.

Missing or ignoring the opportunity to address family emotion can also affect the family-physician partnership because family members may feel unheard or even dismissed. We recognize walking into the emotion is difficult, and it may seem counterintuitive to invite deeper emotions, such as mourning or overt sadness; however, we also know emotional evolution may be necessary for rational decision making.^26,27,28,29^ Shared decision making may therefore necessitate emotional processing.

Families need to feel cared for by their physician.^9^ They want their physicians to show empathy,^30^ and studies suggest that perceptions of physician empathy translate to perceptions of physician competence.^31^ Incorporating empathy into communication has been linked to patient satisfaction, better health outcomes, and reduced physician burnout.^21,32,33,34,35^

In our care conferences, there was always another member of the health care team present. Although they contributed to less than 5% of the dialogue, when they addressed a family’s emotions, these other health care team members were more likely to use unburied empathetic statements offering support to families. These results suggest that maximizing the expertise in the full health care team may offer an additional layer of support in responding to families, and future care conferences should consider increasing the verbal contributions of other health care team members.

Our results offer practical guidance for physicians to consider when communicating with families. Using NURSE statements and then stopping to allow time for a response helps to connect with families and share their experience. Limiting medical talk, using open-ended questions to explore emotions, and reducing physician-to-physician interruptions can provide opportunities to learn new information about patients and their families. Most importantly, listening to patients and their families allows physicians to avoid missing opportunities to deepen the discussion.

### Limitations

The strength of this article lies in a relatively large sample of audio-recorded care conferences with a diverse group of physicians and clinical decisions being discussed with families of critically ill children. Despite the strengths, there are limitations. As a single-center study, the use of empathetic statements may be unique to this group of physicians. We attempted to mitigate this effect by limiting the number of audio recordings per physician and including physicians from many pediatric disciplines. Excluding non-English–speaking families limits our ability to generalize results to all families in the PICU. We excluded conversations about medical updates or discharge planning to focus on high-stakes emotional discussions. Physicians’ patterns of empathetic statements may be different in these other types of discussions. We also recognize that we did not conduct a multivariate analysis of this relationship, and there may be other important confounders, such as family member sex and relationship to patient, which are not addressed. We captured only formal, scheduled family-physician conversations and recognize we cannot comment on other opportunities to communicate with families, such as during bedside meetings and family-centered rounds.

## Conclusions

While physicians frequently responded with empathy, their responses were often buried within medical talk. When physicians use transparent, unburied empathetic statements to respond to family emotion, it leads to a deeper conversation and can reveal a family’s fears, values, and motivations.
